# Circadian Potency Spectrum in Light-Adapted Humans

**Published:** 2022-08-18

**Authors:** Martin Moore-Ede, Anneke Heitmann

**Affiliations:** 1 Circadian Light Research Center, Stoneham, MA, USA;; 2 Korrus Inc, Los Angeles, CA, USA

**Keywords:** Circadian, Light, Spectral Sensitivity, Human

## Abstract

Light exposure at night can disrupt the circadian timing of cellular processes and is associated with a broad range of health disorders. To spectrally engineer lighting which minimizes circadian disruption at night it is necessary to define the precise spectral sensitivity of the human circadian system. Prior attempts have used short monochromatic light exposures in dark-adapted human subjects, or *in vitro* dark-adapted isolated retina or melanopsin. However, humans spend virtually all their awake hours in a fully light-adapted state. Here we review the evidence for a narrow blue circadian sensitivity curve for light-adapted humans derived from experiments using spectral filtering of light sources, and comparisons of light sources with diverse spectral power distributions. This light-adapted Circadian Potency function permits the development of circadian-protective light for nocturnal use and circadian-entraining light for daytime use.

## INTRODUCTION

Previous assessments of the spectral sensitivity of the circadian system, based on *in vivo* and *in vitro* studies have shown spectral sensitivity across a broad range of wavelengths, including violet (400–429 nm), blue (430–499 nm) and green (500–560 nm) wavelengths. These studies used dark-adapted subjects with induced pupil dilation [1–3], dark-adapted pieces of human retinae [4] and melanopsin in Human Embryonic Kidney Cells (HEK-293) [5], and the light exposures were relatively short (e.g., 30 min [1], 90 min [2,3]). These derived spectral sensitivity curves peaked between 457–480 nm [2], which is distinct from the spectral sensitivity peaks of other retinal photoreceptors (rod: 498 nm; S-cone: 420 nm; M-cone: 534 nm; L-cone: 564 nm) [4].

However, the test conditions of the ‘dark-adapted’ light-exposure studies are very different than the typically much longer (up to 16+ hours), continuous and fully-adapted exposure of humans to polychromatic white light in everyday life. Because of the initial dark adaptation, the previously reported circadian spectral sensitivity curves may include, in addition to 430–499 nm (blue) wavelength sensitivity, transient 400–429 nm (violet) and 500–560 nm (green) components resulting from cone- and rod-mediated extrinsic inputs to Intrinsically-Photoreceptive Retinal Ganglion Cells (ipRGCs) which decay over the first two hours of extended light exposure.

In experiments with longer (6-hour) exposure to monochromatic blue or green light (460 nm or 555 nm, respectively), conducted with initially dark-adapted subjects [6], both monochromatic lights equally suppressed melatonin over the initial 30 minutes, but blue light maintained its suppressive effects across the 6-hour exposure time, while melatonin suppression with green light disappeared within two hours after its initial effects. This initial green-light sensitivity may be attributed to potential transitory extrinsic input to ipRGCs from M-cones and does not have a significant impact on the circadian spectral sensitivity curve for light-adapted conditions [6].

Transient effects may be also seen with violet light exposure, resulting from transitory extrinsic input to ipRGCs from S-cones. Experiments on previously dark-adapted subjects showed that melatonin suppression by monochromatic violet light (420–424 nm) was much larger after 30 minutes (95% suppression) [1] than after 90 minutes (27% suppression) [3]. In another experiment, only minimal melatonin suppression was seen after three hours of exposure to light with violet (420 nm) peak emission [7].

## LITERATURE REVIEW

### Discovery of light-adapted human circadian sensitivity curve

The light-adapted human circadian spectral sensitivity curve under normal workplace lighting conditions has now been defined using a) light exposure times of greater than 12 hours instead of brief ≤90 minute light exposures of the dark-adapted eye; b) polychromatic overhead ceiling white light sources, instead of monochromatic lights; c) exposures to illumination levels recommended by the IES Lighting Handbook for work surfaces (300–1,000 lux, horizontally at tabletop) [8]; and d) measurements of the total 12-hour nocturnal (20:00 h to 08:00 h) suppression of melatonin (area under the curve) as a circadian disruption biomarker [9] that has direct relevance to health outcomes [10].

**Figure 1:**
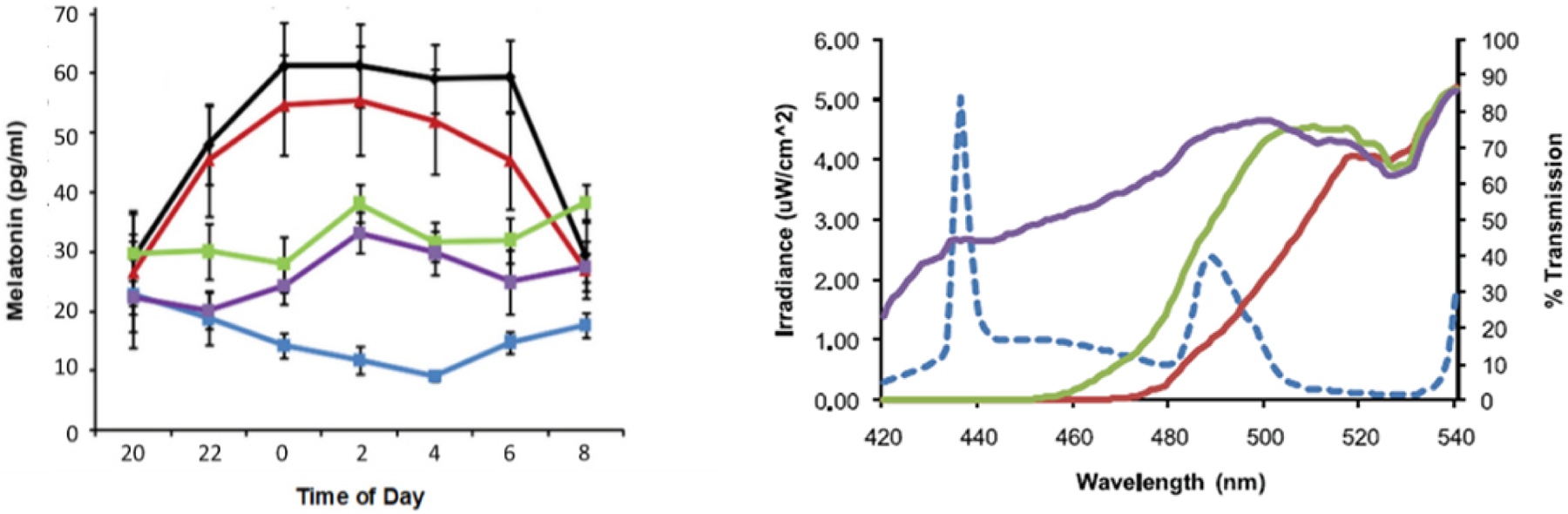
Left: Salivary melatonin levels in pg/mL (mean ± SEM) measured overnight (20:00 h to 08:00 h) from subjects in darknesss (black), unfiltered light (light blue), filtered light with <485 nm cut off filter (red), <470 nm cut-off filter (green) and a placebo light filter (purple). Right: Spectral irradiance of unfiltered light source (dashed line) and percent transmission curves of <485 nm cut off filter (red), <470 nm cut-off filter (green) and placebo filter (purple). With permission from [11].

The first indication that the light-adapted circadian sensitivity curve was narrower than the published dark-adapted curve came from studies using eyeglasses with dichroic cut-off filters (Figure 1). In human subjects illuminated overnight by polychromatic white light for 12 hours at workplace levels of light intensity (600–1000 lux tabletop), the melatonin circadian rhythm which normally peaks in the early morning hours is heavily suppressed [11]. But there was near complete restoration of melatonin to levels comparable to those during darkness when <485 nm dichroic cut off filters (<10% transmission) were used to eliminate violet and blue, but not green wavelengths [11,12]. In contrast, when <470 nm dichroic cut off filters (<10% transmission) were used, partial suppression of melatonin occurred indicating that blue wavelengths between 470–485 nm contributed significantly to melatonin suppression, but there was also suppressive activity in blue wavelengths less than 470 nm.

**Figure 2:**
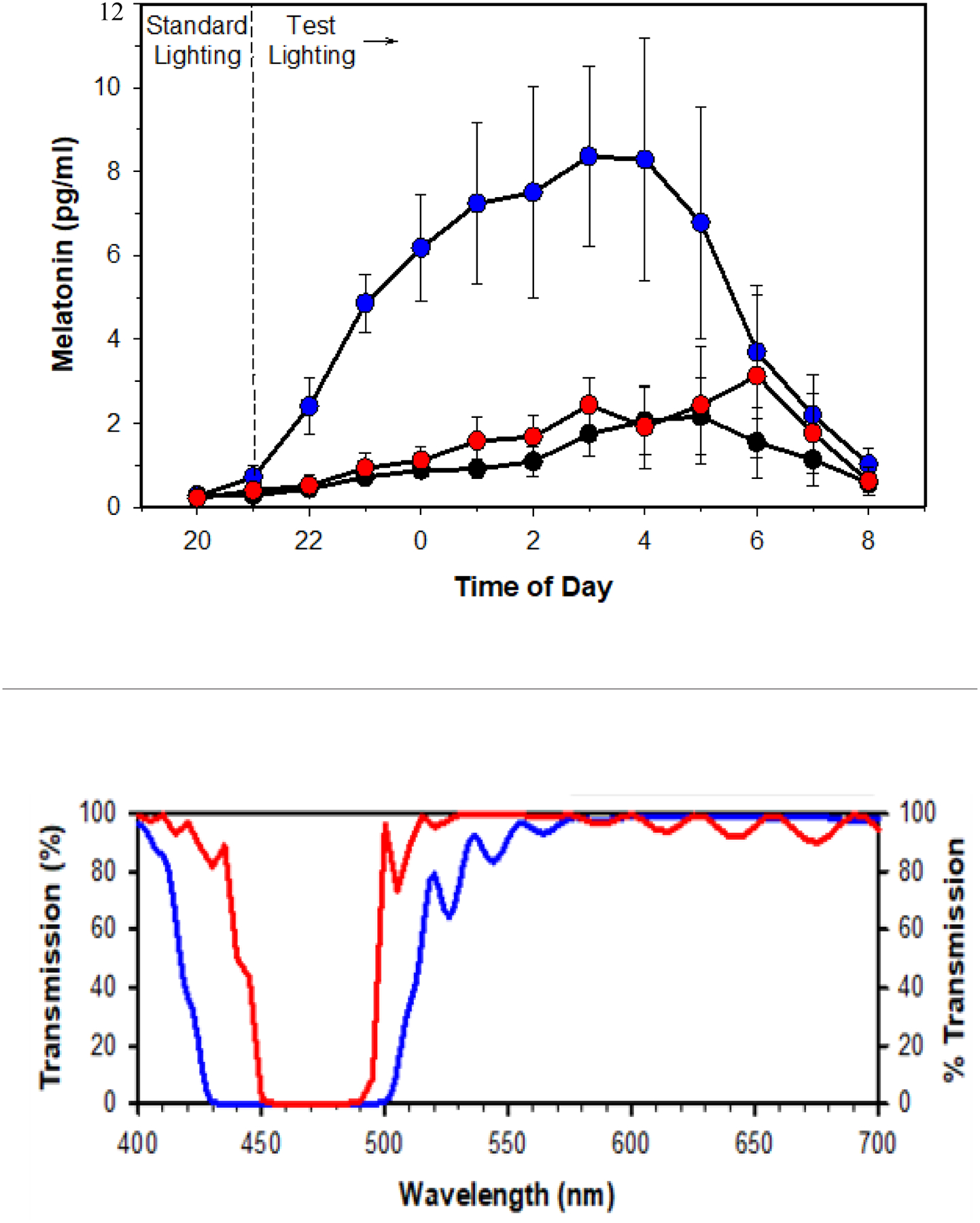
Top: Salivary melatonin levels in pg/mL (mean ± SEM) measured overnight (20:00 h to 08:00 h) from subjects in unfiltered light (black) 450–490 nm filtered light (red) and 430–500 nm filtered light (blue). Bottom: Transmission percent of 450–490 nm notch filter (red) and 430–500 nm notch filter (blue). **Note:** (—) 430–500 nm Notch Filter, (—) 450–490 nm Notch Filter.

We explored the spectral boundaries of light-adapted human circadian sensitivity by comparing human subjects illuminated from 20:00 h to 08:00 h overnight by an unfiltered LED overhead light source providing 540 lux illumination at tabletop, and with the same subjects under the light source with dichroic notch filters which either removed all wavelengths between a) 450–490 nm or b) 430–500 nm. The nocturnal pattern of salivary melatonin was suppressed under the unfiltered light source and partially suppressed by the light source with 450–490 nm filter, but the melatonin curve was unsuppressed with the 430–500 nm filter (Figure 2), indicating that wavelengths between 430 and 460 nm made a significant added contribution to circadian spectral sensitivity.

**Figure 3:**
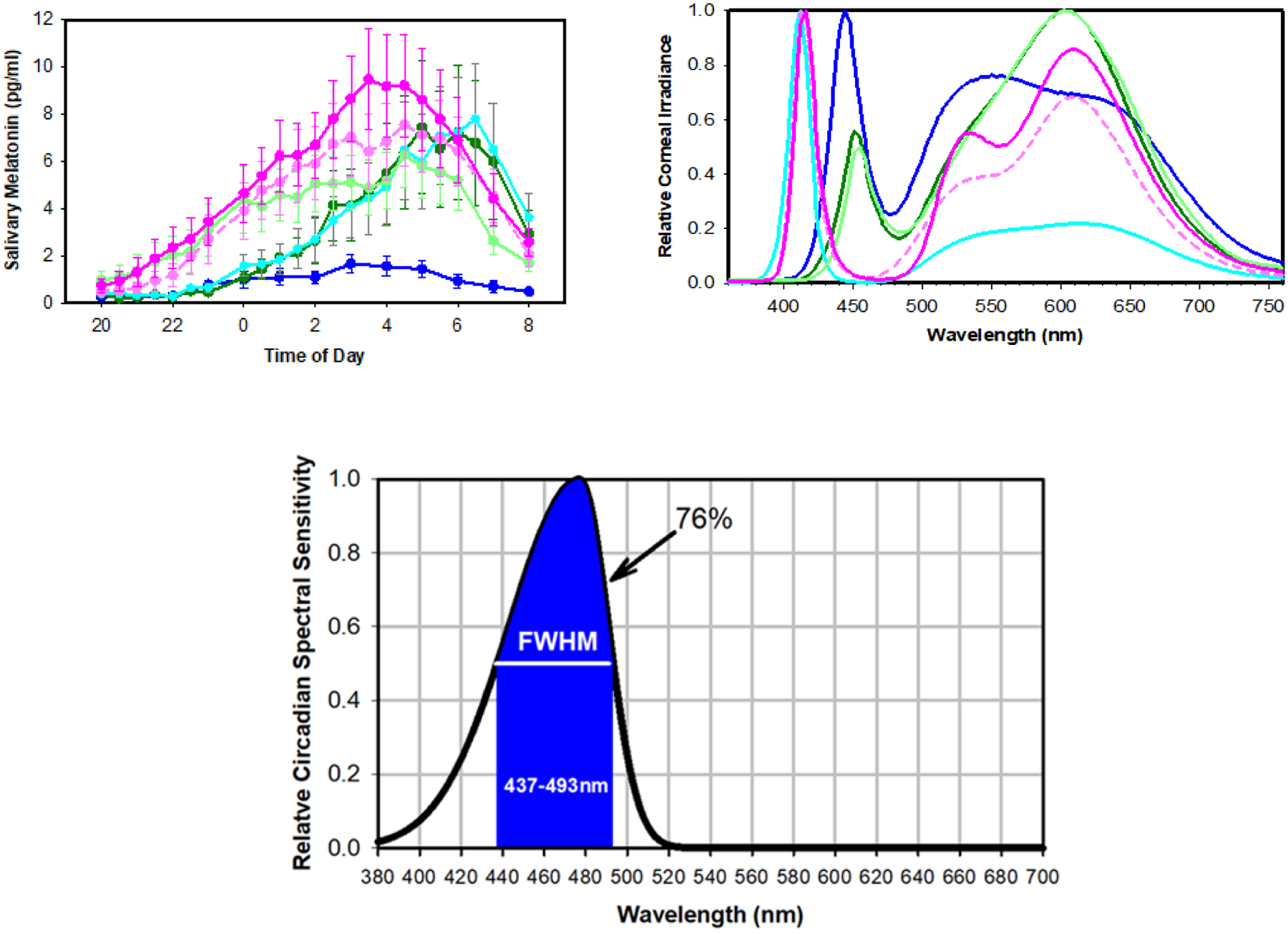
Spectral sensitivity to 12-h exposure to six spectrally-diverse polychromatic white Light-Emitting Diode (LED) light sources (A-F). Top Left: Salivary melatonin levels in pg/mL (mean ± SEM) measured overnight (20:00 h to 08:00 h) from subjects under each LED light source. Top Right: Normalized spectral power distributions of light sources measured at the cornea (eye level) of human subjects seated around a conference table illuminated at 540 lux tabletop. Bottom: Optimized Circadian Potency spectral sensitivity curve that provided the best linear regression fit with 76% of spectral sensitivity falling in the full-width half maximum 438–493 nm blue band depicted with a wavelength peak of 477 nm blue. **Note:** (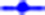) A, (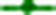) B, (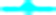) C, (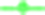) D, (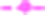) E, (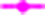) F.

To measure the spectral sensitivity of light-adapted human subjects more precisely, we ran a similar 12-hour overnight protocol, using, instead of filtered light, six spectrally diverse polychromatic white LED light sources (A-F). Based on the resulting nocturnal salivary melatonin patterns, a steady-state human spectral sensitivity curve was derived. This Circadian Potency spectral sensitivity curve with a Full Width at Half Maximum (FWHM) of 438–493 nm and peaking at 477 nm [13] is much narrower than the previous ‘dark-adapted’ curves (Figure 3).

The Circadian Potency peak wavelength of 477 nm, obtained from light-adapted subjects under extended polychromatic light exposure, is close to the *in vitro* peak sensitivity (479 nm) of human melanopsin [5], indicating that the sustained light-adapted circadian response is mediated by ipRGC melanopsin. The Circadian Potency peak wavelength also supports findings by Rüger et al. [14] that showed that low-irradiance (12 μW/cm^2^) monochromatic blue light (480 nm) near that Circadian Potency peak wavelength generates an amplitude of the circadian phase response curve that is comparable to that of a polychromatic light source with much higher irradiance level (3000 μW/cm^2^).

## DISCUSSION

The human circadian timing system can be disrupted by non-optimal light exposure such as insufficient day light or excessive evening or night light. Light at night is associated with detrimental health effects including compromised mood, sleep and immunity, higher obesity and diabetes risks and higher rates of neuroendocrine sensitive cancers such as breast and prostate cancer [15–17]. Consequently, night shift work with circadian disruption was classified as a “probable (group 2A) human carcinogen” by the World Health Organization International Agency for Research on Cancer (IARC) [18], and the US National Toxicology Program expert panel designated excessive light at night and insufficient light during the day as “reasonably anticipated to be a human carcinogen” [19].

Defining the specific spectral characteristics of harmful and helpful electric lighting by means of spectral sensitivity curves is important to help spectrally engineer appropriate lighting solutions. The Circadian Potency spectral sensitivity curve [13] has several practical advantages in comparison to the sensitivity curves obtained from the earlier experiments with short exposure of dark-adapted subjects to monochromatic light [1–3]:

1. The Circadian Potency curve is applicable to real-world situations as it was obtained under typical workplace conditions with light-adapted subjects and extended exposure to polychromatic light.
2. The Circadian Potency spectral sensitivity curve spans a narrower wavelength range (FWHM 438–493 nm) than the rather broad (400–560 nm) range of the ‘dark-adapted’ spectral sensitivity curves that spread across violet, blue and green wavelengths. Removing this wide wavelength range from the polychromatic (380–780 nm) white light spectrum for the purpose of healthy nocturnal lighting is not practical for manufacturing attractive white or near-white light desired for most human purposes.
3. Further, the narrower Circadian Potency spectral sensitivity curve makes it feasible to engineer energy-efficient nocturnal lighting using energy efficient violet LED dies which would not be feasible according to the ‘dark-adapted’ sensitivity curve.

The fact that the Circadian Potency spectral sensitivity curve suggests relative insensitivity at spectral wavelength below 425 nm is of particular interest:

1. It allows the use of violet (410–425 nm) LED dies to engineer calorimetrically-balanced white light for nocturnal lighting instead of the conventionally used blue LEDs with ~450 nm peak emissions.
2. The replacement of blue wavelengths by violet wavelengths reduces the negative health impacts of blue-rich nocturnal lighting, while maintaining the positive effects of short wavelength light on alertness and performance [20,21], and there is evidence that the alerting effects of violet light (420 nm) are even greater than seen with blue (440 nm or 470 nm) light [22].

Besides using the Circadian Potency spectral sensitivity curve for spectrally designing healthy nocturnal white lighting, it can be also used for applications to phase shift the circadian clock for purposes of adjusting to shift work or jetlag, or for treating circadian sleep disorders. While specific Phase Response Curves (PRCs), that describe how wavelengths with high Circadian Potency can produce phase delays or phase advances, are not determined, the closest approximation currently available is the type 1 PRC that was developed for monochromatic blue (480 nm) light [14]. The question of why the circadian system is only spectrally sensitive to a narrow band of blue wavelengths led us to postulate an evolutionary connection. Early life originated deep in the oceans, where all visible light except for wavelengths of ~475 nm are absorbed by seawater below 200 meters depth [23]. Life forms have thus long relied on blue wavelengths to receive day-night signals and synchronize circadian clocks. This predictive adaptation to the earth’ rotation [24] is seen in humans as well as unicellular marine organisms such as Gonyaulax [25], and is expressed in the circadian machinery of a) blue photoreceptors with maximal sensitivity at ~ 475 nm, b) circadian clocks, and c) nocturnal darkness-promoted melatonin production [26].

## CONCLUSION

Under normal light-adapted conditions with polychromatic white light, the spectral sensitivity of the human circadian system appears to be limited to a narrow band of blue wavelengths with a peak at 477 nm and a FWHM of 438–493 nm. This Circadian Potency function has a narrower sensitivity range than the spectral sensitivity curves previously derived from short exposures to monochromatic lights under dark-adapted conditions. This appears to be because violet and green light effects seen in dark-adapted subjects are transient components that decay with extended exposure to light. The light-adapted Circadian Potency spectral sensitivity function has enabled the development of sensors and spectrally-engineered LED light sources to minimize circadian disruption. It also makes it possible to address the health risks of light exposure at night in our 24/7 society, by alternating between daytime circadian-entraining white light spectra and nocturnal circadian-protective white light spectra without loss of visual acuity.
